# Heatwave Definition and Impact on Cardiovascular Health: A Systematic Review

**DOI:** 10.3389/phrs.2023.1606266

**Published:** 2023-10-16

**Authors:** Julia Nawaro, Lorenzo Gianquintieri, Andrea Pagliosa, Giuseppe M. Sechi, Enrico Gianluca Caiani

**Affiliations:** ^1^ Department of Electronics, Information and Bioengineering, Politecnico di Milano, Milan, Italy; ^2^ Agenzia Regionale Emergenza Urgenza (AREU), Milan, Italy; ^3^ Istituto Auxologico Italiano IRCCS, Milan, Italy

**Keywords:** heat, extreme temperature, cardiovascular health, mortality, morbidity

## Abstract

**Objectives:** We aimed to analyze recent literature on heat effects on cardiovascular morbidity and mortality, focusing on the adopted heat definitions and their eventual impact on the results of the analysis.

**Methods:** The search was performed on PubMed, ScienceDirect, and Scopus databases: 54 articles, published between January 2018 and September 2022, were selected as relevant.

**Results:** In total, 21 different combinations of criteria were found for defining heat, 12 of which were based on air temperature, while the others combined it with other meteorological factors. By a simulation study, we showed how such complex indices could result in different values at reference conditions depending on temperature. Heat thresholds, mostly set using percentile or absolute values of the index, were applied to compare the risk of a cardiovascular health event in heat days with the respective risk in non-heat days. The larger threshold’s deviation from the mean annual temperature, as well as higher temperature thresholds within the same study location, led to stronger negative effects.

**Conclusion:** To better analyze trends in the characteristics of heatwaves, and their impact on cardiovascular health, an international harmonization effort to define a common standard is recommendable.

## Introduction

Due to climate change, extreme weather events such as heatwaves, drought, hurricanes, and floods are becoming more frequent worldwide [1–3]. The years 2015–2021 were reported as the warmest 7 years period since 1850 [4]. Furthermore, apart from their frequency, heatwaves are also increasing in intensity and duration [5, 6], with their climatological characteristics related to rainfall and weak pressure gradient events [7, 8]. The Intergovernmental Panel on Climate Change identified the risk of death and illness from heatwaves, especially in the vulnerable population of urban areas, as one of the eight major risks related to global climate change [9].

The negative impact of high temperatures on human health has been assessed by several studies [10–12]. In particular, passive heat stress can affect the human cardiovascular (CV) system by increasing the heart rate and left ventricular contractility, and by reducing central blood volume, left ventricular filling pressures and cerebral perfusion [13]. Furthermore, people with a pre-existing CV disease have been found more vulnerable to high temperature, as it reduces the organism’s ability to thermoregulate [14]. A recent systematic review and meta-analysis provided evidence of the increased CV mortality and morbidity in heat conditions based on past data [15]. Also, other aspects were explored in literature, from the projected effects of heat on health under different climate change scenarios [16–20], to the effectiveness of heat warning systems in decreasing mortality [21].

However, despite the abundance of studies on heat effects on CV health, surprisingly, there is not a unique definition of heat or heatwave, which potentially hinders the comparison of epidemiological studies. Also, the conditions required to trigger an alert in heat warning systems vary by country, both in terms of meteorological indicators and applied thresholds [22]. As a result, this complicates the comparison of epidemiological studies dealing with the potential impact of heat on human health, due to the usage of different temperature (or thermal indices) indicators. Since the beginning of the 20th century, more than 150 thermal climate indices related to human health have been proposed in the scientific literature [23]. Several standardization attempts were performed [24–30], but they failed in the identification of a unique heat definition, even within the same geographical area.

In light of the above considerations, the objective of this study was to conduct a systematic review considering open access papers published in the last 5 years, during which time the attention towards this topic has increased worldwide. The focus of the analysis was on the effects of heat on CV health, primarily considering the applied definition of heat and, secondarily, assessing whether such definitions had an impact on the applied methodology and on the reported results.

## Methods

In order to ensure transparency, reproducibility, and effectiveness, a systematic review approach [31, 32] was applied. The Preferred Reporting Items for Systematic Review and Meta-Analysis (PRISMA) guidelines [33] were followed. Three online databases of scientific literature were examined—PubMed, ScienceDirect, and Scopus—of which the first covers biomedicine and life science, while the other two provide an overview of multidisciplinary research. Databases were queried in the title, abstract, or keywords using as keywords “heat*,” “high temperature*,” “extreme weather,” paired with “cardiovascular,” “heart,” “ischemic,” “cardiac,” “infarction,” “myocardial,” “hypotension,” “hypertension.” Only journal articles written in English, published between January 2018 and September 2022, and with full text open access availability were included.

Additional filtering was performed on the basis of full-text content. First, papers without an explicit definition of heat were discarded. Then, only original studies with analytic study design (i.e., cohort study, case-control study, or case-crossover study) based on official medical records (i.e., registered deaths, hospital admissions, emergency calls) were included, excluding those based on personal perception (i.e., questionnaires). Finally, for comparison purposes, only articles that reported the results as relative risk (RR), odds ratio (OR), or incidence density ratio (IDR) with a 95% confidence interval were kept. The characteristics of the included studies can be described with the following PECO [34] statement: (P) Among humans of all ages, genders and ethnicities, what is the effect of (E) exposure to heat versus (C) non-heat conditions on (O) CV morbidity and mortality.

The analysis of the resulting articles explored the following aspects:

1) Definition of heat, considering in particular three aspects:
  • Indicators: meteorological parameters and measures included in the definition.
  • Methods: statistical method applied for the comparison between exposure groups.
  • Comparison threshold: reference value applied to subdivide exposed and non-exposed groups.

The computed values of different heat indices, in relation to different air temperatures at the reference conditions, were also compared.

2) Main characteristics of the study design, such as the analyzed outcome, medical data source, geographic distribution, sample size, and meteorological data source.
3) Quantification of the impact on CV health, considering the statistical methods and the reported results, in relation to the applied definition of heat.

All-cause CV diseases refer to the International Classification of Diseases codes 390–459 (ICD-9-CM) or I00–I99 (ICD-10-CM). To address disease-specific analyses, the results among six groups of pathologies were grouped: myocardial infarction (ICD-10 code I21–I23), ischemic (coronary) heart disease (including chest pain; I20–I25), stroke (I60–I69), hypertensive diseases (I10–I15), heart failure (I50) and other diseases (i.e., chronic rheumatic heart disease, out-of-hospital cardiac arrest, acute aortic dissection, arrhythmias).

In the following text, the term “heat” is used as abbreviation of “heatwave,” the acronym TEMP is used for “temperature,” while the term “outcome” describes the medical result (i.e., the received diagnosis or death), and “result” refers to the RR/OR/IDR reported in the studies. If a factor results in a “negative effect on health” it means that the risk of developing a health-related negative CV event is increased. All reported plots were created with Plotly library (version 5.11.0) in Python (version 3.8.5).

## Results

**FIGURE 1:**
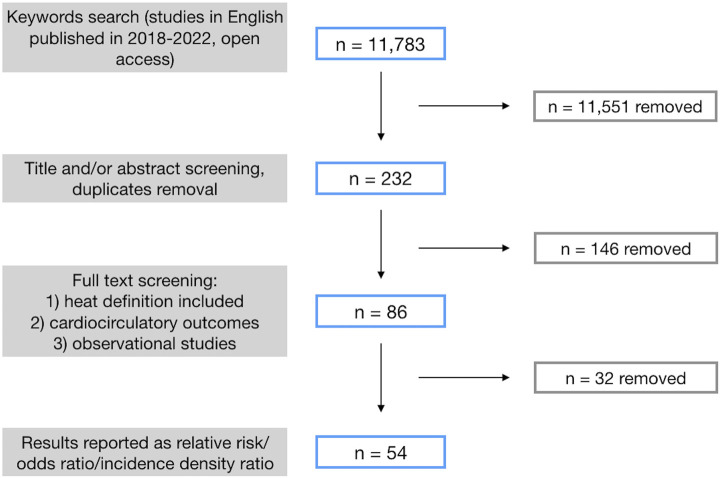
Schematic of the results of the systematic literature review process, including open access papers published between 2018 and 2022, written in the English language, and resulting from the application of the selected query (see text for details) (systematic review, international, 2018–2022).

The adopted query resulted in the identification of 11,783 articles (3,602 in PubMed, 2,390 in ScienceDirect and 5,791 in Scopus) in the English language and published between January 2018 and September 2022. After title and/or abstract screening, 347 items were selected as possibly relevant articles, of which 232 were unique. During the phase of full text screening, 146 studies were excluded as not relevant, due to the lack of an explicit definition of heat, not focusing in the reported results on CV outcomes, or using a study design other than analytic. Lastly, considering only articles reporting results (i.e., the influence of heat on CV health) as RR, OR, or IDR with 95% confidence, 54 articles were finally selected [35–88]. Figure 1 shows the summary of this literature systematic review process.

### Definition of Heat

In total, 21 different combinations of criteria to define heat were found (Table 1), with only three studies simultaneously testing and comparing more than one of them. The most frequently used meteorological measure as heat indicator was air TEMP, applied in 56 of 57 cases. In nine studies, air TEMP was included in the computation of more complex indices, such as “Apparent Temperature” [42, 53, 60, 71, 76, 83], “Perceived Temperature” [39], “Pseudo-equivalent daily temperature” [88] or “Discomfort Score” [56], by combining it with relative humidity, water vapor pressure, dew point TEMP, wind speed and/or heat radiation. The relevant calculation formulas are summarized in Table 2, except for “Perceived Temperature” [39] as it was not provided in the relevant study. Only one study [74] used air masses (excluding air TEMP) to define heat days.

**TABLE 1:** Summary of indicators used to define heat (systematic review, international, 2018–2022).

Indicator	Method	Comparison threshold	Number of articles	References
Air temperature	Percentile	MMT or equivalent	21	[36, 43, 44, 46–51, 55, 57, 62, 66–68, 70, 73, 78, 80, 84, 86]
		Another percentile	4	[42, 44, 64, 79]
		Non-heatwave	3	[58, 59, 85]
		Mean temperature	1	[52]
		Arbitrary absolute value	1	[75]
		Other	1	[61]
	Percentile and duration	Non-heatwave	8	[37, 38, 40, 45, 54, 63, 72, 82]
	Absolute value	MMT or equivalent	1	[87]
		Non-heatwave	1	[38]
	Absolute value and duration	Non-heatwave	4	[35, 65, 77, 81]
	Other, i.e., EHF	Another percentile	1	[69]
		Non-heatwave	1	[41]
Air temperature + relative humidity	Percentile	Another percentile	1	[56]
Air temperature + water vapor pressure	Percentile	Non-heatwave	1	[76]
	Absolute value and duration	Non-heatwave	1	[88]
Air temperature + dew point temperature	Percentile	MMT or equivalent	1	[60]
		Another percentile	1	[71]
Air temperature + relative humidity + wind speed	Percentile	MMT or equivalent	1	[53]
		Another percentile	2	[42, 83]
Air temperature, humidity, wind speed, heat radiation	Absolute value	Non-heatwaves	1	[39]
Air masses	—	Other air masses	1	[74]

MMT, minimum mortality temperature; EHF, Excess Heat Factor. The total is 57 articles as three of 54 studies applied more than one combination of criteria.

**TABLE 2:** Calculation formulas for complex heat indices (systematic review, international, 2018–2022).

Heat index name	Formula	Abbreviations	References
Discomfort Score	$\frac{T+T\_\text{wetbulb}}{2}$	T —air TEMP,	[56]
		T_wetbulb —wet bulb TEMP (derived from air TEMP and relative humidity)	
Pseudo-equivalent daily temperature	T+1.5*VP	T —air TEMP,	[88]
		VP —water vapor pressure	
Apparent Temperature 1	−1.3+0.92*T+2.2*VP	T —air TEMP,	[76]
		VP —water vapor pressure	
Apparent Temperature 2	$T+0.33*\frac{\text{RH}}{100}*6.105*e^{17.27*\frac{T}{237.7+T}}-0.7*\text{WS}-4$	T —air TEMP,	[53], [42], [83]
		RH —relative humidity,	
		WS —wind speed	
Apparent Temperature 3	$-2.653+0.994*T+0.0153*{T\_\text{dewpoint}}^{2}$	T —air TEMP,	[71], [60]
		T_dewpoint —dew point TEMP	

In the majority of cases (*n* = 46), heat days were defined using thresholds on percentiles of TEMP distribution over a certain period, with TEMP defined by daily mean, minimum or maximum values. The three most frequent thresholds were 99th (*n* = 17), 95th (*n* = 13) and 97.5th (*n* = 11) percentiles. In eight cases, the thresholds were set on absolute TEMP values, in the range 20°C–35°C. In several studies, the threshold criterion was coupled with a duration requirement (e.g., TEMP above the threshold for at least 2–5 days). When the study period was limited to the warmer months only, the considered TEMP distribution was either annual or periodic. The lowest value of heat threshold reported in literature was a mean daily temperature of 20°C [39], set in Austria, while the highest was a mean daily temperature of 43°C, set in Kuwait [73].

As regards the threshold-based identification of the non-exposed groups for comparison, this was selected based on:

• the minimum mortality TEMP (MMT) or its equivalent (minimum hospital admission TEMP, minimum ambulance calls TEMP etc.), derived from the relevant minimum mathematical function applied to the temporal series.
• another percentile (i.e., the 50th, 75th, 90th of the TEMP distribution or the 1st percentile of heat index values).
• defining non-heatwave days (i.e., all days not classified as heat).
• mean TEMP or arbitrary absolute value.

Moreover, in one study, instead of a threshold for direct comparison, the authors analyzed the effects of a 1°C increase in TEMP above the selected threshold [61].

It is worth noting that in four studies the heat definitions were used at a national level for heat warning systems (Table 3).

**TABLE 3:** National definitions of heat (systematic review, international, 2018–2022).

Country	Heat definition	References
Australia	EHF ≥ 85th percentile of the distribution of its positive values; where $EHF=\frac{T_{i}+T_{i+1}+T_{i+2}}{3}-T_{95}*\max\left(1,\frac{T_{i}+T_{i+1}+T_{i+2}}{3}-\frac{T_{i}+T_{i-1}+\ldots +T_{i-30}}{30}\right)$ ,	[69]
	where $T_{i}$ is the air TEMP at day i and $T_{95}$ is the 95th percentile of daily mean TEMP distribution at day i . If a heatwave is detected for day i , days i+1 and i+2 are considered a heatwave as well	
Belgium	Daily maximum TEMP > 25°C for at least 5 consecutive days, including at least 3 days with daily maximum TEMP ≥ 30°C	[35]
Latvia	1st level of heat: daily maximum TEMP ≥27°C and <33°C for at least 2 consecutive days	[77]
	2nd level of heat: daily maximum TEMP ≥ 33°C	
Sweden	Low intensity heat: daily maximum TEMP ≥ 30°C for 3 consecutive days	[81]
	High intensity heat: daily maximum TEMP ≥ 30°C for 5 consecutive days and/or daily maximum TEMP ≥ 33°C for 3 consecutive days	

Country column refers to the country in which the definition is used at national level. EHF, Excess Heat Factor as defined in Nairn and Fawcett [89].

**FIGURE 2:**
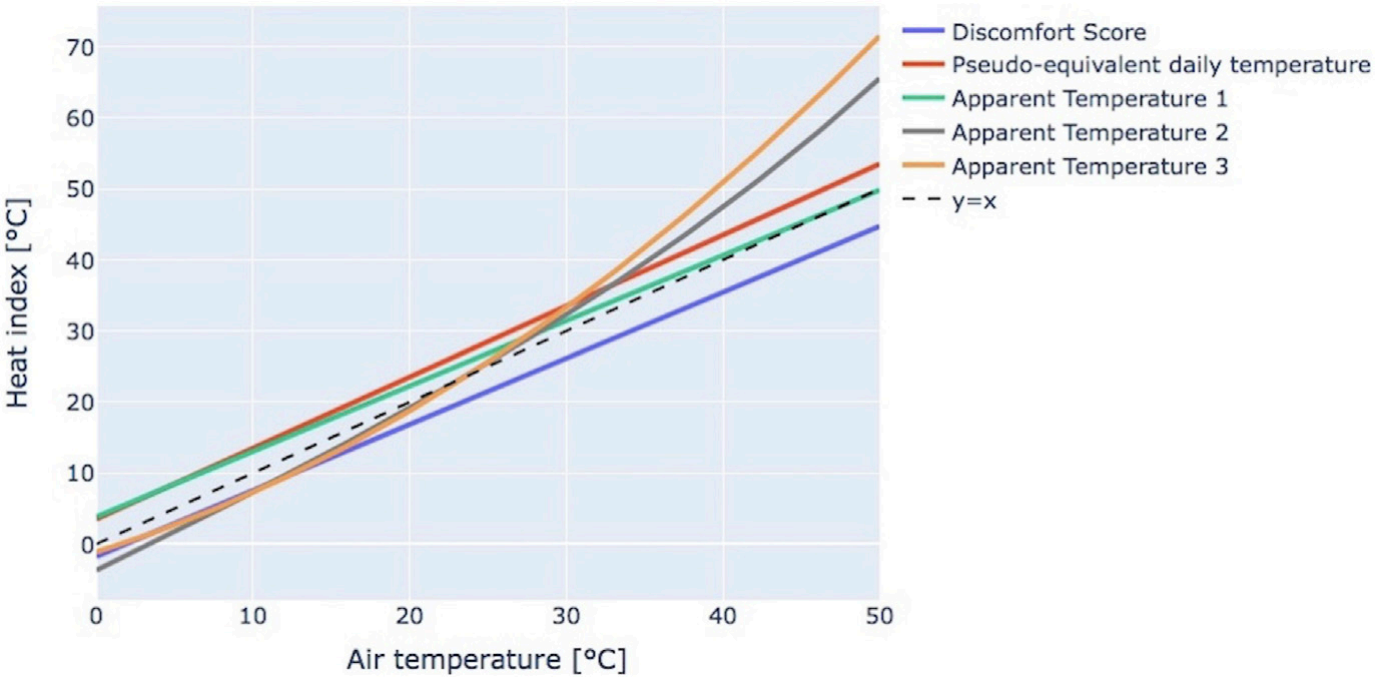
Heat indexes as a function of temperature as defined in Table 2, computed assuming relative humidity = 50%, wind speed = 1 m/s, water vapor pressure = 2.34 kPa and dew point temperature calculated according to Lawrence [90] (systematic review, international, 2018–2022).

For a deeper analysis and visual comparison of the different heat indexes summarized in Table 2, in Figure 2 they were plotted as a function of air TEMP, with the other parameters arbitrarily set to standard conditions (relative humidity = 50%, wind speed = 1 m/s, water vapor pressure = 2.34 kPa, dry bulb TEMP equal to air TEMP and dew point TEMP calculated according to Lawrence [90]).

From this graph, it can be noted that, in colder environments (i.e., 0°C–20°C), Pseudo-equivalent daily temperature and Apparent Temperature 1, based on water vapor pressure, reach significantly higher values compared to the other indexes, with a maximum difference up to 7°C. When the air TEMP is closer to 30°C, which is considered in many studies as the threshold for heat days, there are no big differences among the indexes, except the Discomfort Score, characterized by lower values. Above a value of air TEMP equal to 30.4°C, the differences among indexes increase progressively, with Apparent Temperature 2 and 3 rising to higher values due to their exponential/polynomial formulas.

### Study Characteristics

Among the selected articles, 27 (50%) were targeting the analysis of mortality, 25 (46.3%) of morbidity and 2 (3.7%) of both these outcomes (Supplementary Figure S1).

Among the studies including morbidity as a target, the majority (*n* = 18) referred to hospital admissions (including three on emergency hospital admissions), while the rest considered emergency calls (*n* = 4), emergency department visits (*n* = 3), chest pain centre visits (*n* = 1), or general practitioner consultations (*n* = 1).

In 21 (38.9%) studies, only all-cause CV diagnoses were considered, while in the remaining 33 (61.1%) studies the results were stratified by specific CV disease. Additionally, regardless of the target outcome, in a few studies the impact of heat was also studied on specific subgroups, stratified by age (9 studies; 16.7%), by age adjusted for exposure to noise or chronic conditions (2 studies; 3.7%), or by the presence of a chronic condition alone (1 study; 1.9%).

The geographical origin of the selected studies spanned areas from all six continents, with 25 (46.3%) articles from Asia, 15 (27.8%) from Europe, 8 (14.8%) from North America, 3 (5.6%) from Oceania, 2 (3.7%) from South America and 1 (1.9%) from Africa. The country covered with the highest number of studies was China, using either data from single cities [42, 52, 53, 65, 75, 83, 87], individual provinces [46, 48, 68], or multiple cities spread throughout the country [37, 47, 86].

The sample size of the target study group was explicitly reported in 40 (74.1%) articles; alternatively, it was inferred by multiplying the average daily incidence by the study period when both characteristics were specified in the article (*n* = 10; 18.5%); in 4 (7.4%) studies it was not possible to infer any information about sample size. The median sample size was slightly above 40,000 subjects, with a minimum of 939 individuals in a study about congestive heart failure hospital admissions and mortality in Boston, New York and Philadelphia [85], and a maximum of 1,154,896 in an analysis of all-cause CV morbidity in South Africa [84]. Sample size distribution in the different geographical areas is reported in Supplementary Figure S2.

The median observation period was 10 years, with a minimum of 2 years for Israel [56], Slovenia [88] and a Chinese study [83], and a maximum of 28 years for Germany [57]. Several studies (*n* = 22; 40.7%) analyzed the influence of heat only in an arbitrarily defined warmer period of the year, beginning within March–June and ending within June–October for the Northern Hemisphere, while spanning from September-November to March for the Southern Hemisphere. When narrowing the study period, the most frequent interval was May-September (*n* = 9; 16.7%).

Regarding the use of meteorological data as explicative attributes in the analysis, dew point/air TEMP, relative humidity, wind speed, atmospheric/barometric pressure, precipitation, sunshine duration, solar radiation and air pollution were utilized. When the mean values for each factor were reported (*n* = 36; 66.7%), they were calculated by averaging all records from all measurement stations in the study area. In 9 (16.7%) studies, interpolated meteorological maps (e.g., maps at ZIP code level [82], district level [84] or at 2 km spatial resolution [36]) were alternatively used.

Among the 13 (24.1%) studies for which the events’ location or patients’ home address were available, only two [48, 56] associated them with the meteorological conditions at the event time, while Jiang et al. [86] linked the TEMP with that at the hospital location, as the majority of patients did not report their complete address.

### Quantification of Heat Impact on Cardiovascular Health

To assess the impact of heat on CV health, the majority of articles (*n* = 39; 72.2%) applied the distributed lag non-linear model, which describes the response to exposure accounting for a delayed effect [91]. The number of tested lags varies up to 30 days, with the most frequent choices being 21 (*n* = 15; 27.8%), 3 (*n* = 9; 16.7%), and 14 (*n* = 5; 9.3%).

Six studies considered the duration of the temperature increase as a criterion to classify a heatwave, but only in two studies was a stronger negative effect reported. Other applied techniques included the generalized linear models (*n* = 13; 24.1%), or a simplified approach based on the comparison between incidence rates during heat and non-heat days (*n* = 2; 3.7%). In several studies, mostly in those using only air TEMP to define heat, the statistical models were further adjusted by meteorological factors (*n* = 9; 16.7%), by air pollution (*n* = 4; 7.4%) or by both (*n* = 22; 40.7%).

In 14 studies (25.9%) a case-crossover design, as proposed by Maclure [92], was applied. By using the same subjects as both cases and control at different points in time, this design allows to control for time-invariant characteristics, such as gender or medical history.

In the majority of studies, the results were reported as RR (*n* = 44; 81.5%), and in the remaining studies as OR (*n* = 7; 13%) or IDR (*n* = 3; 5.6%). The values of these three indicators are approximately equal when the initial risk (i.e., prevalence of the disease in the population under study in non-heat conditions) is relatively small [35, 93, 94], and are therefore comparable under this assumption.

Most of the results (*n* = 37, 68.5%) showed a negative influence of heat for all-causes CV health (i.e., an increase in risk in relation to the comparison threshold as indicated in Table 1) in the range from 1.02 in Spain (95% CI: [1.00–1.04]) [70] and New York State (95% CI: [1.01–1.04]) [58] to 1.47 (95% CI: [1.43–1.51]) in Jiangsu Province, China [48], with two outliers equal to 4.61 (95% CI: [3.67–5.78]) [69] and 3.09 (95% CI: [1.72–5.55]) [73]. However, the studies differed in the applied heat definition (Table 1). All studies that reported a negative effect of heat on CV health found this effect stronger if associated with a shorter lag, while this relation seemed to weaken over time. In disease-specific analysis, the most consistent and significant results were reported for stroke, varying from 1.20 (95% CI: [1.02–1.40]) [79] to 1.62 (95% CI: [1.39–1.88]) [46], with the most frequently associated heat definition based on the combinations of air TEMP, percentile, and MMT equivalent. The strongest disease-specific result was found for myocardial infarction (5.22, 95% CI: [2.14–12.73]), with heat defined using Apparent Temperature 2, percentile and MMT equivalent [53].

However, in 11 studies (20.4%) no statistically significant results were found, while in 6 (11.1%) a statistically significant decrease in the risk of developing a CV acute event during heat was surprisingly reported. Of these six, one study analyzed all CV diseases together [83], while the others referred to specific diseases, i.e., myocardial infarction [79], hypertensive diseases [76, 79], heart failure [45, 63, 79], and acute aortic dissection [75].

A complete summary of these results is presented in Supplementary Figure S3 for all-cause CV outcomes and in Supplementary Table S1 for specific CV diseases.

Finally, to identify a possible correlation between the applied definition of heat and the reported results relevant to the CV risk, an additional analysis was performed considering the percentage deviation of the reported heat threshold from the mean annual temperature, calculated as:

$$100*\;\frac{heat\;threshold\;–\;mean\;annual\;temperature}{mean\;annual\;temperature}$$

**FIGURE 3:**
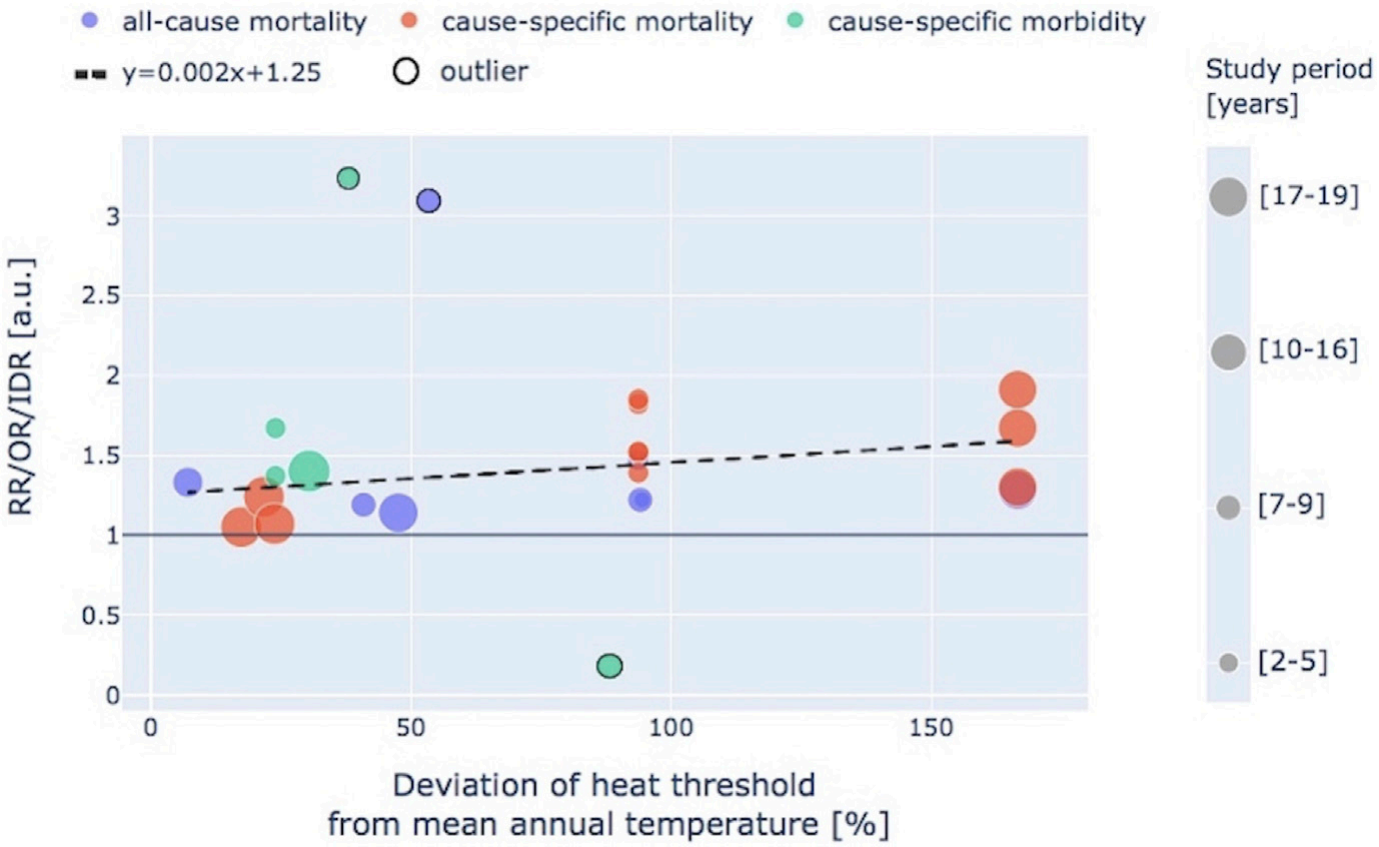
Relationship between the percentage deviation of the considered heat threshold from the mean annual temperature and associated cardiovascular risk, subdivided as all-cause and cause-specific mortality or morbidity. RR, relative risk; OR, odds ratio; IDR, incidence density ratio. Dot size is proportional to the duration of the observation period expressed as number of years (systematic review, international, 2018–2022).

This computation was feasible in 13 (24.1%) studies in which the absolute values of both parameters were explicitly specified, and relevant results are presented in Figure 3. Excluding three outliers (i.e., all-cause CV mortality in Kuwait [70], out-of-hospital cardiac arrest events in Israel [56], and acute aortic dissection morbidity in China [75]), a slight trend of higher risk with increasing deviation of the heat threshold from the mean annual temperature could be observed, yet not with robust statistical evidence, with the coefficient estimated by ordinary least squares linear regression equal to 0.002 (*R* ^2^ = 0.19).

## Discussion

In this systematic review, studies focusing on the impact of heat on CV morbidity and/or mortality published in the last 5 years were selected, in order to assess the latest progresses made by the scientific community in this field. The identified studies were conducted across all six continents, with a leading role of Asia (both in terms of number of publications and of sample size), followed by Europe and North America. Source data spanned from 1987 to 2021.

Despite having the same goal, the examined study settings were extremely heterogeneous. Such heterogeneity could be noticed both in the definition of heat in the field of CV health, but also in other aspects, such as the medical and meteorological data used, the sample size, the observation periods, and the applied methodology for analysis. In addition, biases due to measurement error [95] or spatial autocorrelation [96] could affect data generation and processing.

### Applied Definitions of Heat

As early as the beginning of the 21st century, Robinson [25] pointed out that, due to the lack of an unequivocal heat definition, it was difficult to assess if heatwaves varied in intensity or prevalence, which further hindered research into climate change. The majority of heat indices developed since the beginning of the 20th century have been collected and organically presented in a theoretical framework [23]. However, to the best of our knowledge, a comparison of their viability in health-related studies is still missing. Moreover, only a small fraction of those indices uses exclusively meteorological factors, such as air TEMP, relative humidity and solar radiation [97] or air TEMP, dewpoint TEMP, relative humidity, cloud cover, and wind speed [98], whereas many other indices (e.g., [99–103]) are also based on human thermal comfort factors (i.e., human metabolic rate), a type of data that is labor-intensive in terms of collection and processing, thus hindering their application in heat-health related research. In the examined recent literature, only one review focusing on heat definitions and addressing their heterogeneity was found [104]. It included 60 studies published in 2001–2015 covering Asia, Australia, Europe, and North America, considering the impact of heat on all-cause CV and respiratory mortality.

In line with this established knowledge, twenty-one unique combinations of indicators, methods, and comparison thresholds, with the purpose of defining heat days and to distinguish them from non-heat days, were identified in the studies selected for this review. Differently from de Freitas and Grigorieva [23], the majority of these studies used air TEMP alone, averaging the values obtained from measurement stations without applying spatial interpolation. Other meteorological data (i.e., relative humidity, water vapor pressure, dew point TEMP, wind speed and/or heat radiation) were eventually used only for a following adjustment of the statistical models.

Several different approaches could be identified:

- even when a combined indicator was applied, it is worth noting that three different versions of “Apparent Temperature” were found, as a result of the application of simplified versions, depending on data availability, to the same original index [23].
- only the term “Perceived Temperature” can be referred directly to a specific and standardized heat index.

The choice of the index to be considered for the analysis is strongly relevant in terms of comparing results obtained in different studies. In fact, as shown in Figure 2, the values of the different indexes as a function of TEMP cannot be assumed to be the same, except for a narrow range around 30°C.

Two main methods for setting the heat threshold were identified, namely, using a percentile of the indicator’s distribution or its absolute value. However, in this latter case, the reported threshold values varied in a range of 15°C, making it impossible to formulate a universal threshold with this strategy. In addition, four national definitions implemented in heat warning systems were identified, one of which applied to a territory different from the country in which the criterion was defined. Our findings, showing strong heterogeneity in heat definitions applied to research studied published from 2018 to 2022, indicated that despite the increased international interest in climate change effects on health, there was no important progress in the harmonization and standardization process towards a singular heat definition to be applied in the context of CV health. A similar finding was already present in a former systematic review including 60 studies published in the period 2001–2015 [104], thus further highlighting the lack of action of the scientific community towards this problem.

It is important to assess if there is an overall trend, transversal to different settings, in the relationship between the selected definition of heat and the related results in terms of impact on CV health. In this perspective, Xu et al. [104] noted that a modification of the threshold value led to a more important change in mortality than the modification of heatwave duration, which is consistent with our findings. In studies where multiple thresholds were tested, a higher risk was usually reported for higher thresholds. This trend is in agreement with Xu et al. [105], who focused on heat definitions and five health events (i.e., ambulance service uses, all-cause and cause-specific emergency department attendances, all-cause and cause-specific hospitalizations) in Brisbane, Australia. In that study, multiple heat definitions were tested, combining three durations (2–4 days) and 10 percentiles (90th–99th) applied on mean and maximum air TEMP. The authors concluded that, for three of these five health events, a threshold selection with the 97th percentile resulted in a significant association with heat (regardless of duration and TEMP indicator), while for the other two a significant correlation was found with a threshold set to 98th or 99th percentile (depending on TEMP indicator). Among the studies included in this review, it is worth noting that two outliers with very elevated health risk in all-cause CV events were identified for Seville and Kuwait. The former compared the 99th percentile of a heat index distribution, accounting for the TEMP in time windows of up to 30 days, to its 1st percentile. The latter compared the 99th percentile of daily mean air TEMP distribution to the MMT. In both geographical areas, a very high percentile threshold was considered even though both of these territories are characterized by particularly hot summers, thus focusing on extreme events only, in a set-up that could partly explain the outlier results.

### Impact of Heat on Cardiovascular Health

As a secondary target, this review also focused on the results of the analysis conducted in the included studies, with an aim of assessing the impact of heat on CV health, thus addressing the applicability and robustness of heat definitions towards this specific purpose.

On this topic, Xu et al. [104] concluded, pooling the results of their review, that an increased mortality could be found in the heat condition. Moreover, in the United States, the relationship between temperature and all-cause mortality was found to be non-stationary from a spatial point of view [106]. In previous research on the effect of heat on mortality and morbidity, both individual (e.g., age, gender, medical history, race, education level, occupation) and community-level (e.g., housing quality, medical care, air pollution) characteristics were identified as effect modifiers [107–112]. In this regard, the case-crossover method should present an advantage over traditional cohort or case-study designs, as it overcomes the between-person confounding factors. In the studies here considered, the vast majority of results reported a negative impact of heat on all-cause CV health, yet they used different parameters (mainly RR, but also OR and IDR) computed from different data sources (hospital admissions, emergency calls, emergency visits). In general, no specific information on the patient’s home address could be matched with the meteorological conditions at the time of the event.

In CV disease-specific analysis of both morbidity and mortality, an increased risk for stroke and ischemic (coronary) heart disease was evidenced in all the examined studies. In the case of the former, similar results have been previously reported, being particularly strong for ischemic stroke [113, 114], with possible explanations including dehydration, increased blood viscosity, hemoconcentration, and elevated cholesterol levels during heat [113]. In addition, indications that a sudden and large increase in TEMP (i.e., ∆TEMP ≥ 5°C) increases the risk of ischemic stroke events, rather than the absolute TEMP values, were given [115].

The increased risk of coronary artery disease could be explained with endothelial dysfunction expressed as a reduction of flow-mediated vasodilation associated with increased temperature [116]. When patients are exposed to a hot environment, the CV system increases the cardiac output to augment blood flow to the skin for cooling [117], thus increasing the heart oxygen consumption and potentially generating a precipitation of events towards myocardial infarction. An increased risk for this pathology was reported in 11/12 analyzed studies. Similarly, a previous study demonstrated that higher environmental temperatures were associated with an increased risk in myocardial infarction [118], with an influential effect during the first 6 h after exposure.

The increased risk found in 5/7 studies on hypertension-related outcomes may seem contradictory as a response to heat, but it was explained with a possible interaction between heat and anti-hypertensive medications [79].

When focusing on heart failure morbidity, a lower risk was apparently associated with heat. However, one of these studies also analyzed heart failure mortality and reported a higher RR. This finding is in agreement with other reviews on the effects of heat on CV diseases, where a predominance of negative effects was reported for mortality, in contrast with an impact on morbidity that was not always consistent [15, 119–121].

Among other causes, risk of aortic dissection was found to be reduced with heat, while risks of out-of-hospital cardiac arrest, chronic rheumatic heart disease, and arrythmias were found to be increased.

### Study Limitations and Conclusion

Our study has some limitations. Despite the selection of studies being conducted with the most uniform approach possible with regards to the methodology, the heterogeneity of resulting articles made any advanced meta-analysis unfeasible. The dissimilarities did not only concern the main target of this work, which was the definition of heat, but also affected other factors as well, such as the health outcomes or the characteristics of the studied population. The attempt to generalize the results, neglecting details such as the considered effect modifiers, may have led to some particular trends being missed. Moreover, studying heat and heatwaves has consistent and complex climatological implications that were not considered in detail in this review, as the focus was limited to the effects of heat on cardiovascular health. Lastly, an inherent limitation of our study is that it considered only open access publications over an arbitrarily selected short time frame of 5 years. However, this choice was made to focus on more recent studies to capture the state-of-the-art relevant to the topic, as a previous systematic review by Xu et al. (2016) covered previously published studies. As the literature search and data extraction were performed by a single person, this constitutes a possible methodological limitation of our approach.

Despite the abundance of recent research in this field, there is no single definition of heat, together with a possible confusion introduced by the terminology and related different formulas for the utilized heat indices. An international effort should be conducted in order to harmonize a common standard in representing and evaluating such data including the definition of heatwave. This would allow better comparison among geographical areas and analysis of trends in heatwave intensity, duration and prevalence, as well as the human heat adaptation capabilities. Such analyses could be incorporated into public health programs with a two-fold strategy: short-term intervention, by developing automated systems capable of analyzing and predicting, based on the current environmental variables on a given territory, the possible immediate impact of heatwaves, to guide emergency services in a better organization and deployment of available resources, as well as activating heat alert systems; and mid-long term intervention, by highlighting those features for a certain territory that could influence the citizen’s resilience, and planning urbanistic interventions accordingly.

## Supplementary Material

Supplementary Figure S1 Summary of articles by target outcome (systematic review, international, 2018–2022).

Supplementary Figure S2 Summary of selected articles divided by continent of interest for the analysis, stratified by sample size of the examined study group; in 10 (18.5%) studies, the sample size was inferred considering the average incidence and the study period (systematic review, international, 2018–2022).

Supplementary Figure S3 Summary of the influence of heat on all-cause cardiovascular outcomes. The notation e in the last column means that the sample size was inferred as not directly specified in the relevant study. The letters a–l refer to the combination of criteria used to define heat: a – air TEMP + percentile + MMT or equivalent, b – air TEMP + percentile and duration + non-heatwave, c – air TEMP+relative humidity+wind speed + percentile + MMT or equivalent, d – air TEMP + percentile + non-heatwave, f – air temperature + dew point temperature + percentile + MMT or equivalent, g – air TEMP + percentile + another percentile, h – air TEMP + absolute value and duration + non-heatwave, i – air TEMP + other + another percentile, j – air TEMP+relative humidity+wind speed + percentile + another percentile, k – air TEMP + absolute value + MMT or equivalent, l – air TEMP+water vapor pressure + absolute value and duration + non-heatwave (systematic review, international, 2018–2022).

Supplementary Table S1 Summary of the influence of heat on cause-specific cardiovascular outcomes. The notation e in the “Sample size” column means that the sample size was inferred as not directly specified in the relevant study (systematic review, international, 2018–2022).
