# Supplementary material for: Heatwave Definition and Impact on Cardiovascular Health: A Systematic Review
Source: Public Health Rev. 2023 Oct 16;44:1606266. doi: 10.3389/phrs.2023.1606266 (PMC10613660; doi:10.3389/phrs.2023.1606266)
Supplement: Supplementary file 1 [file Table1.docx]

**Supplementary Table S1** Summary of the influence of heat on cause-specific cardiovascular outcomes. The notation *e* in the last column means that the sample size was inferred as not directly specified in the relevant study (systematic review, international, 2018–2022).

| **Study** | **RR/OR/IDR** | **95% CI** | **Sample size** | **Heat definition (indicator+method+comparison threshold)** |
| --- | --- | --- | --- | --- |
| **Myocardial infarction** | | | |  |
| Mortality | | | |  |
| Saucy et al. (35) | 1.67 | (1.09 - 2.55) | 3248 | air TEMP + percentile + MMT or equivalent |
| Xu et al. (48) | 1.52 | (1.42 - 1.62) | 169,886 | air TEMP + percentile + MMT or equivalent |
| Ma et al. (46) | 1.40 | (1.22 - 1.60) | 98,516 *e* | air TEMP + percentile + MMT or equivalent |
| Ferreira et al. (49) | 1.24 | (1.16 - 1.32) | 118,898 | air TEMP + percentile + MMT or equivalent |
| Ferreira et al. (49) | 1.07 | (1.00 - 1.15) | 131,394 | air TEMP + percentile + MMT or equivalent |
| Ferreira et al. (49) | 1.05 | (1.03 - 1.08) | 118,898 | air TEMP + percentile + MMT or equivalent |
| Morbidity | | | |  |
| Li et al. (53) | 5.22 | (2.14 - 12.73) | 3161 | air temperature + relative humidity + wind speed + percentile + MMT or equivalent |
| Layton et al. (82) | 1.53 | (1.00 - 2.34) | 608 | air TEMP + percentile and duration + non-heatwave |
| Dang et al. (43) | 1.36 | (1.06 - 1.73) | 3328 | air TEMP + percentile + MMT or equivalent |
| Chen et al. (57) | 1.14 | (1.00 - 1.29) | 27,310 | air TEMP + percentile + MMT or equivalent |
| Lam et al. (51) | 1.14 | (1.00 - 1.31) | 53,769 | air TEMP + percentile + MMT or equivalent |
| Schulte et al. (79) | 0.88 | (0.82 - 0.95) | 68,861 | air TEMP + percentile + another percentile |
| **Ischemic (coronary) heart disease (incl. chest pain)** | | | |  |
| Mortality | | | |  |
| Xu et al. (48) | 1.52 | (1.45 - 1.60) | 295,829 | air TEMP + percentile + MMT or equivalent |
| Ma et al. (46) | 1.49 | (1.32 - 1.68) | 164,980 *e* | air TEMP + percentile + MMT or equivalent |
| Saucy et al. (36) | 1.30 | (1.04 - 1.62) | 10,521 | air TEMP + percentile + MMT or equivalent |
| Huang et al. (40) | 1.22 | (1.13 - 1.21) | 25,753 *e* | air TEMP + percentile and duration + non-heatwave |
| Chen et al. (47) | 1.19 | (1.11 - 1.28) | 306,601 | air TEMP + percentile + MMT or equivalent |
| Yin et al. (37) | 1.17 | (1.05 - 1.30) | 306,601 | air TEMP + percentile and duration + non-heatwave |
| Oudin et al. (81) | 1.10 | (1.01 - 1.20) | 68,299 *e* | air TEMP + absolute value and duration + non-heatwave |
| Morbidity | | | |  |
| Li et al. (53) | 1.99 | (1.12 - 3.51) | 8006 | air temperature + relative humidity + wind speed + percentile + MMT or equivalent |
| Lin et al. (78) | 1.37 | (1.00 - 1.89) | 4455 *e* | air TEMP + percentile + MMT or equivalent |
| Guo et al. (59) | 1.22 | (1.11 - 1.34) | N/A | air TEMP + percentile + non-heatwave |
| Moghadamnia et al. (71) | 1.09 | (1.02 - 1.19) | 15,094 | air temperature + dew point temperature + percentile + another percentile |
| Li et al. (58) | 1.07 | (1.03 - 1.11) | 64,262 | air TEMP + percentile + non-heatwave |
| Bai et al. (44) | 1.06 | (1.01 - 1.11) | 1,389,057 | air TEMP + percentile + MMT or equivalent and air TEMP + percentile + another percentile |
| Parry et al. (45) | 1.04 | (1.00 - 1.08) | 68,334 | air TEMP + percentile and duration + non-heatwave |
| Li et al. (53) | 1.01 | (1.00 - 1.11) | 14,447 | air temperature + relative humidity + wind speed + percentile + MMT or equivalent |
| **Stroke** | | | |  |
| Mortality | | | |  |
| Ma et al. (46) | 1.62 | (1.39 - 1.88) | 311,032 *e* | air TEMP + percentile + MMT or equivalent |
| Chen et al. (47) | 1.41 | (1.26 - 1.59) | 415,227 | air TEMP + percentile + MMT or equivalent |
| Xu et al. (48) | 1.39 | (1.31 - 1.47) | 412,567 | air TEMP + percentile + MMT or equivalent |
| Yin et al. (37) | 1.21 | (1.04 - 1.40) | 415,227 | air TEMP + percentile and duration + non-heatwave |
| Schulte et al. (79) | 1.20 | (1.02 - 1.40) | 15,296 | air TEMP + percentile + another percentile |
| Morbidity | | | |  |
| Alsaiqali et al. (35) | 1.45 | (1.04 - 2.03) | N/A | air TEMP + absolute value and duration + non-heatwave |
| Jiang et al. (76) | 1.26 | (1.01 - 1.48) | 70,380 *e* | air temperature + water vapor pressure + percentile + non-heatwave |
| **Hypertensive diseases** | | | |  |
| Mortality | | | |  |
| Saucy et al. (36) | 1.91 | (1.20 - 3.06) | 2728 | air TEMP + percentile + MMT or equivalent |
| Ma et al. (46) | 1.89 | (1.41 - 2.54) | 40,435 *e* | air TEMP + percentile + MMT or equivalent |
| Xu et al. (48) | 1.85 | (1.68 - 2.04) | 75,557 | air TEMP + percentile + MMT or equivalent |
| Schulte et al. (79) | 1.18 | (1.02 - 1.38) | 17,142 | air TEMP + percentile + another percentile |
| Morbidity | | | |  |
| Li et al. (58) | 1.04 | (1.01 - 1.07) | 220,058 | air TEMP + percentile + non-heatwave |
| Jiang et al. (76) | 0.99 | (0.98 - 0.99) | 1,502,460 *e* | air temperature + water vapor pressure + percentile + non-heatwave |
| Schulte et al. (79) | 0.72 | (0.64 - 0.81) | 29,533 | air TEMP + percentile + another percentile |
| **Heart failure** | | | |  |
| Mortality | | | |  |
| Schulte et al. (79) | 1.22 | (1.05 - 1.43) | 14,753 | air TEMP + percentile + another percentile |
| Morbidity | | | |  |
| Hopp et al. (63) | 0.94 | (0.90 - 0.98) | 19,498 | air TEMP + percentile and duration + non-heatwave |
| Parry et al. (45) | 0.83 | (0.76 - 0.90) | 24,721 | air TEMP + percentile and duration + non-heatwave |
| Schulte et al. (79) | 0.83 | (0.76 - 0.90) | 57,440 | air TEMP + percentile + another percentile |
| **Other (i.e. chronic rheumatic heart disease, out-of-hospital cardiac arrest, acute aortic dissection, arrhythmias)** | | | |  |
| Mortality | | | |  |
| Xu et al. (48) | 1.82 | (1.31 - 2.51) | 8171 | air TEMP + percentile + MMT or equivalent |
| Morbidity | | | |  |
| Kranc et al. (56) | 3.24 | (1.90 - 5.86) | 12,401 | air temperature +relative humidity + percentile + another percentile |
| Lin et al. (78) | 1.67 | (1.23 - 2.26) | 4583 *e* | air TEMP + percentile + MMT or equivalent |
| Wang et al. (80) | 1.40 | (1.25 - 1.57) | 100,254 *e* | air TEMP + percentile + MMT or equivalent |
| Li et al. (58) | 1.06 | (1.01 - 1.11) | 81,290 | air TEMP + percentile + non-heatwave |
| Parry et al. (45) | 1.06 | (1.00 - 1.12) | 32,682 | air TEMP + percentile and duration + non-heatwave |
| Yu et al. (75) | 0.18 | (0.01 - 0.55) | 2120 | air TEMP + percentile + arbitrary absolute value |
