# Supplementary figures and images for: Heatwave Definition and Impact on Cardiovascular Health: A Systematic Review
Source: Public Health Rev. 2023 Oct 16;44:1606266. doi: 10.3389/phrs.2023.1606266 (PMC10613660; doi:10.3389/phrs.2023.1606266)

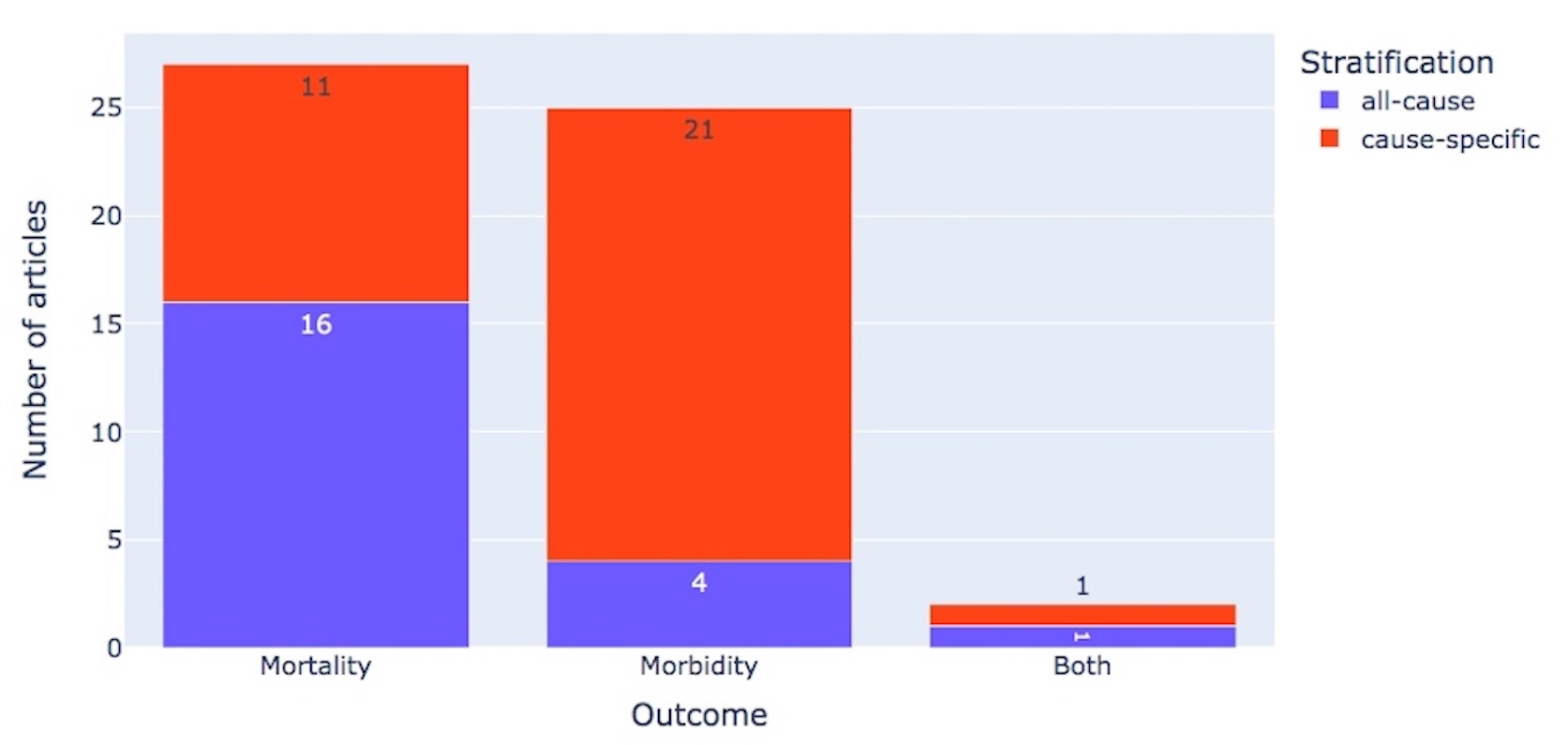

Supplement: Supplementary file 2 [file Image1.JPEG]

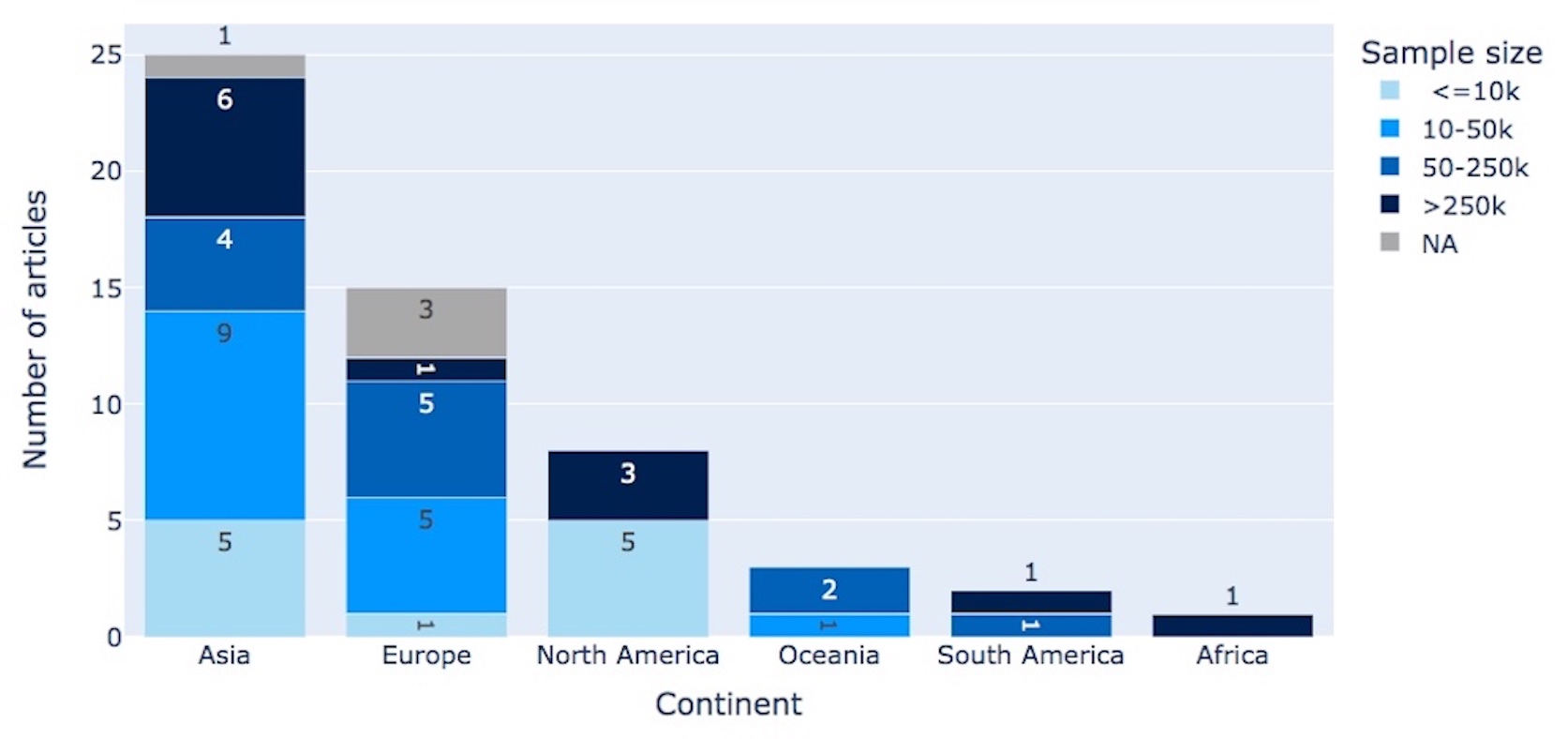

Supplement: Supplementary file 3 [file Image2.JPEG]

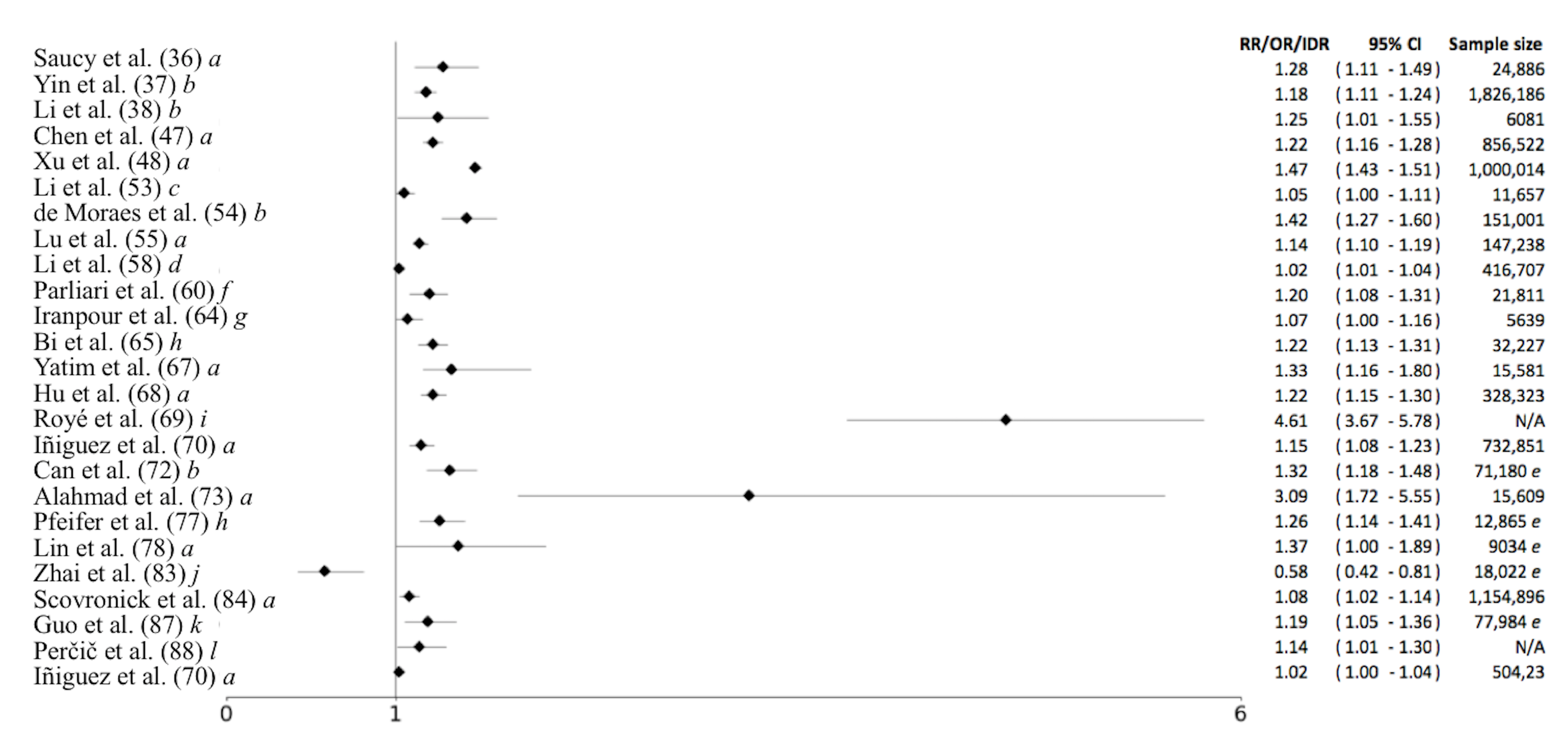

Supplement: Supplementary file 4 [file Image3.PNG]
